# Hand, Foot, and Mouth Disease in China: Critical Community Size and Spatial Vaccination Strategies

**DOI:** 10.1038/srep25248

**Published:** 2016-04-29

**Authors:** Thomas P. Van Boeckel, Saki Takahashi, Qiaohong Liao, Weijia Xing, Shengjie Lai, Victor Hsiao, Fengfeng Liu, Yaming Zheng, Zhaorui Chang, Chen Yuan, C. Jessica E. Metcalf, Hongjie Yu, Bryan T. Grenfell

**Affiliations:** 1 Department of Ecology and Evolutionary Biology, Princeton University, Princeton, NJ, USA; 2 Institute of Integrative Biology, ETH Zurich, Zurich, Switzerland; 3 Division of Infectious Diseases, Key Laboratory of Surveillance and Early–warning on Infectious Disease, Chinese Center for Disease Control and Prevention, Beijing, China; 4 School of Public Health, Taishan Medical College, Shandong, China.; 5 Department of Geography and Environment, University of Southampton, Southampton, UK; 6 Woodrow Wilson School of Public and International Affairs, Princeton University, Princeton, New Jersey, USA; 7 Fogarty International Center, National Institutes of Health, Bethesda, MD, USA

## Abstract

Hand Foot and Mouth Disease (HFMD) constitutes a considerable burden for health care systems across China. Yet this burden displays important geographic heterogeneity that directly affects the local persistence and the dynamics of the disease, and thus the ability to control it through vaccination campaigns. Here, we use detailed geographic surveillance data and epidemic models to estimate the critical community size (CCS) of HFMD associated enterovirus serotypes CV-A16 and EV-A71 and we explore what spatial vaccination strategies may best reduce the burden of HFMD. We found CCS ranging from 336,979 (±225,866) to 722,372 (±150,562) with the lowest estimates associated with EV-A71 in the southern region of China where multiple transmission seasons have previously been identified. Our results suggest the existence of a regional immigration-recolonization dynamic driven by urban centers. If EV-A71 vaccines doses are limited, these would be optimally deployed in highly populated urban centers and in high-prevalence areas. If HFMD vaccines are included in China’s National Immunization Program in order to achieve high coverage rates (>85%), routine vaccination of newborns largely outperforms strategies in which the equivalent number of doses is equally divided between routine vaccination of newborns and pulse vaccination of the community at large.

Hand, Foot, and Mouth Disease (HFMD) was first identified in New Zealand in 1957^1^, and subsequently has been reported across the Asia-Pacific region where it is now endemic. Over the last two decades, HFMD outbreaks have been reported in: Malaysia^2^, Taiwan, China^3^, Japan^4^, Singapore^5^, Vietnam^6^, Mainland China^7^,^8^, Hong Kong Special Administrative Region China^9^, South Korea^10^, and Cambodia^11^. The disease is mainly caused by multiple serotypes of the Enterovirus A species in the genus Enterovirus of the Picornaviridae family, and predominantly affects young children under the age of five. In most instances, the infected individuals exhibit mild and self-limiting infections that include fever and skin eruptions on the hands and feet, as well as mouth sores^1^. However, in a small proportion of cases (0.03%^12^)—the majority involving the serotype EV-A71—the infection can lead to serious neurological complications that can be fatal^2^,^13^,^14^. Because of these potential complications, HFMD constitutes a considerable burden for health care systems throughout Asia and also has non-negligible economic consequences^15^,^16^.

In China, which accounts for the majority of cases worldwide^17^, up to 11.8 million cases have been reported between 2008 and 2014. In reaction, the Chinese Center for Disease Control and Prevention initiated a surveillance program to reference HFMD cases with an unprecedented level of geographic detail (province, prefecture, county and township levels, respectively 1^st^, 2^nd^, 3^rd^, and 4^th^ administrative divisions). Concomitantly, three manufacturers in China have been developing inactivated monovalent EV-A71 vaccines that were found to provide high protective efficacy (90.0–97.4%) against EV-A71 in infants and young children in phase III trials^18^,^19^. Currently one monovalent EV-A71 vaccine is in license approval process, and two monovalent vaccines were licensed in December 2015 by the Chinese Food and Drug Administration^20^. In this context, a quantitative understanding of the spatio-temporal dynamics of HFMD in China could help to optimally deploy this new vaccine in light of the demographics and the economics of the country as well as the epidemiology of the disease (Wu *et al.*,^21^ in review).

Mathematical models have provided useful insights into the dynamics of similar self-immunizing childhood infections such as measles^22^,^23^,^24^,^25^, rubella^26^,^27^,^28^, or pertussis^29^,^30^,^31^. With regards to vaccination, models can be used to evaluate the impact of different vaccination strategies, and optimally allocate a limited number of vaccine doses across geographic areas^28^ and age groups^32^,^33^. Previous model-based analyses have explored the seasonal patterns of HFMD and attempted to estimate key epidemic indicators such as the basic and effective reproductive numbers^34^,^35^,^36^,^37^. Additionally, Takahashi *et al.*^38^, recently used a time series Susceptible-Infectious-Recovered (TSIR) model to evaluate the potential for competitive release of CV-A16 for a hypothetical nation-wide vaccination campaign against EV-A71. Another important epidemic parameter to characterize in the context of vaccination campaigns is the critical community size (CCS)—the size of an isolated population below which a pathogen cannot persist without re-introduction^39^.

The CCS is a complex parameter that is specific to each disease and depends on the seasonality and the intensity of transmission^40^,^41^, the pattern of arrival of infected individuals, as well as the recruitment of susceptible individuals, either through variations in birth rate or migration^42^. In a context of high vaccination uptake, the CCS can strongly influence the spatio-temporal dynamics and hence the persistence of childhood disease in the long term. As China displays important geographic gradients in population density and in transmission intensity of HFMD^38^, characterizing the CCS across regions could affect the efficiency of competing spatially heterogeneous control strategies, allowing, for example, for reduced focus in coverage on zones below the CCS^43^. To our knowledge, none of the previous modelling studies on HFMD have attempted to quantify the CCS of the disease. The unprecedented spatial resolution of the Chinese surveillance dataset opens new possibilities to investigate the dynamics of HFMD across a range of community sizes and quantify its potential for local persistence.

## Objectives

In this study we use detailed geographic surveillance data to quantify the CCS of HFMD in China’s northern and southern regions using both observed and simulated incidence from TSIR epidemic models. Subsequently, we explore what spatial patterns of vaccination may best reduce the burden of HFMD in a context of limited vaccine availability.

## Methods

### Data

In this study, we used a four-year time series (2010–2013) of weekly incidence of clinically and laboratory confirmed cases of HFMD from the nationwide Notifiable Infectious Diseases Reporting Information System (NIDRIS) maintained by the Chinese Center for Disease Control and Prevention. These sparse time series data were available at the township level (4^th^ administrative division of China) and were aggregated to the county level (3^rd^ administrative division) and to the province or municipality level (1^st^ administrative division) for consistency with Takahashi *et al.*^38^. Weekly incidence of HFMD was also available at the prefecture level but was not used as too few time series experienced interruptions that could be exploited to evaluate the CCS. To reconstruct the respective incidence of EV-A71 and CV-A16 associated HFMD in each province, we used the proportions of each of those serotypes amongst lab-confirmed cases (other serotypes were not considered in this study). This procedure is identical to Takahashi *et al.*^36^ using the data from NIDRIS. At the province level, there were moderate discrepancies between the aggregated incidence of CV-A16 and EV-A71 and the incidence from NIDRIS used by Xing *et al.*^12^ and Takahashi *et al.*^38^ (median provincial correlation = 0.65). At the county level, the incidence time series was reconstructed for each EV-A71 and CV-A16 using binomial sampling of all clinically confirmed HFMD cases, with a sampling probability equal to the proportion of cases associated with either EV-A71 or CA-A16 in the surrounding province. In this study all weekly incidence reports were pooled in years of 52 weeks for simplicity.

### Estimation of critical community size (CCS)

The CCS for EV-A71 and CV-A16 in China was estimated from the county-level incidence in the southern and northern epidemic regions of China (as defined in Xing *et al.*^12^). For each strain we use two approaches to estimate the CCS: first using the observed incidence (corrected for underreporting), and second using simulated incidence from TSIR simulations^23^,^24^,^44^ with immigration dynamics. CCS estimates from township-level incidence were not considered because in most locations, the time series were too sparse to apply the TSIR models. Underreporting rates were obtained for each province by estimating the weekly value of the slope of the relationship between the cumulative number of cases and the cumulative number of births^39^.


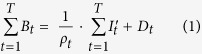


Where *B*_*t*_ is the weekly number of births, *ρ*_*t*_ is the reporting rate estimated using a smoothing spline with four degrees of freedom, 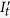 is the observed incidence, and *D*_*t*_ represents the residuals of the fitted function at each time step (Appendix I). The numbers of births by province per week were derived from the yearly birth rates obtained from the National Bureau of Statistics of China. Because the reporting rates obtained were very low, we used a resampling procedure described in Appendix II that prevents underestimating of the tails of each epidemic wave when correcting the incidence for underreporting in small populations. Weekly births at the county level were calculated as the number of weekly births at the province level multiplied by the ratio between each county’s population and its corresponding province population.

#### CCS from reported incidence

The CCS values obtained from the observed incidence (corrected for underreporting) were estimated using the relationship between the log10 of the population in each district and the proportion of zeros in each incidence time series. For each epidemic region and strain, the intersection of the fitted smoothing splines (df = 4, see Appendix IV for sensitivity analysis) with the population axis gives the estimate of the CCS^29^. The population estimate can be interpreted as the threshold population size below which the disease cannot sustain itself without repeated re-introduction

#### CCS from simulated incidence

Next we simulated the weekly incidence of EV-A71 and CV-A16 in each county using a time series SIR model. The dynamics of the susceptible and infectious individuals within each spatial unit are given by


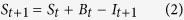



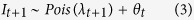



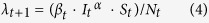


Here, S_t_ is the number of individuals that are susceptible, B_t_ is number of births, I_t_ is the number of infected individuals corrected for underreporting at time t, λ_t+1_ is the expected value of the number of individuals infected at time t + 1, θ_t_ is the number of infected individuals immigrating in the spatial unit, β_t_, is a seasonally-varying transmission rate, α is a correction parameter accounting for non-seasonal heterogeneities in mixing as well as for time discretization^23^,^44^, and N_t_ is the total population size in the spatial unit. The transmission rates β_t_, and the number of migrants θ_t_ were fitted independently in each spatial unit (Eqs 6 and 7) whilst α was spatially invariant. For consistency with previous modelling works on childhood infections, we assumed frequency-dependent transmission, a choice motivated by the fact that for such childhood infections the social clique size is relatively constant^23^,^45^,^46^.

In this study we used the TSIR framework in a two-step procedure: For the first step, we fitted the model at the province level, omitting the immigration term, θ, as its influence on the dynamics is negligible in the very large populations that make up entire Chinese provinces. This allows us to estimate the seasonally varying transmission rates *β*_*t*_ by taking the log transformation of Eq. 4:


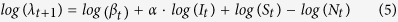


where α is fixed to 0.95^38^, and *S*_*t*_ can be expressed as 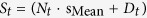 with *D*_*t*_ estimated as the residuals of the fitted smoothing spline of the cumulative number of births on the cumulative number of cases (Eq. 1). The proportion of susceptible individuals s_Mean_ for each province was taken from Takahashi *et al.*^38^.

**Figure 1:**
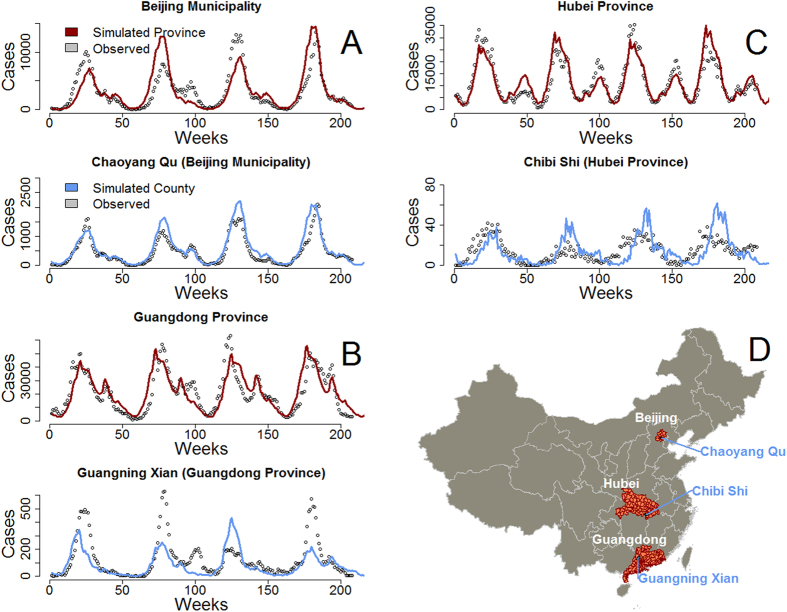
Scaling of the transmission parameters for the TSIR model between the province and county scale in Beijing municipality. (**A**) Guangdong Province (**B**) and Hubei Province (**C**). In each location the average transmission rate 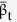 was respectively scaled by a factor of 1.10, 2.42 and 0.05 for A, B and C after re-fitting. This figure was generated using the open source statistical software R (cran.r-project.org), version number 3.0.3, including packages maptools and foreign.

In the second step, we use Eqs 2 and 3 to simulate the dynamics in each of the 2,897 counties that reported HFMD cases and we apply the following scaling factors φ_β,_ and φ_θ_, with values specific to each individual county, on the provincial transmission rate *β*_*t*_ and the immigration term θ_*t,*_ such as,


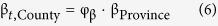


and


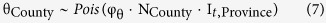


where N_County_ and I_t,Province_ are respectively the number of individuals in the county and the incidence in the province containing the county (Fig. 1). For each county, we ran a constrained minimization procedure with 500 simulations varying the scaling factors within a range of plausible values, ϕ_θ_ = [0, 1] and ϕ_β_ = [0, 10] (varying evenly across the corresponding range), the latter to enable the model to capture potential variation in the intensity of the frequency-dependent transmission (Fig. 1).

The best set of scaling factors was identified by comparing four years of simulated incidence to the observed incidence (corrected for underreporting) using the square mean root error as the fitting metric. Simulations were run for 40 years to bring the system to its dynamic equilibrium, before comparison with the observed incidence time series.

**Figure 2:**
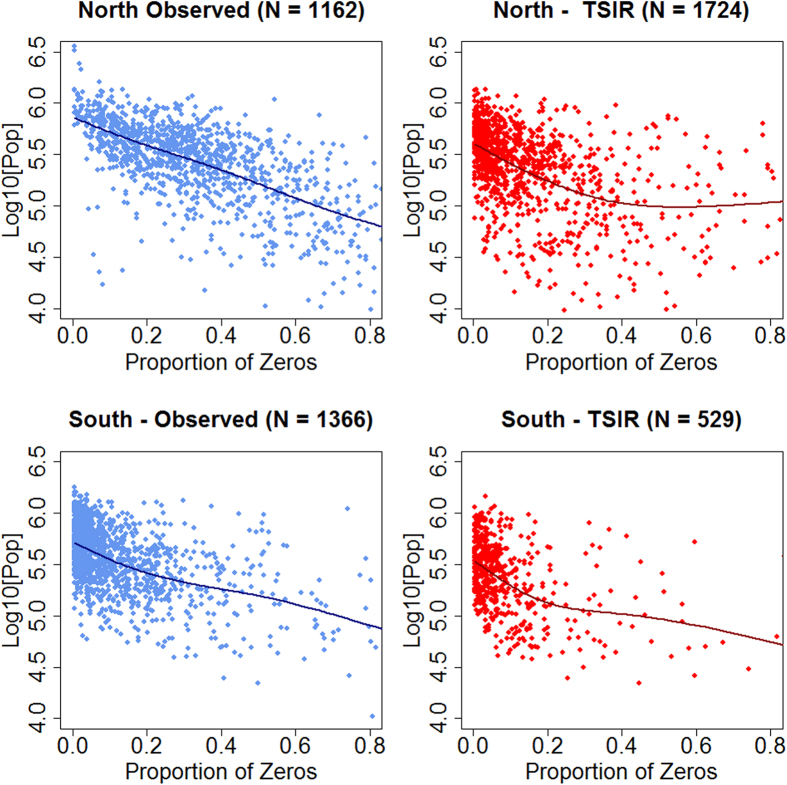
Critical Community size of EV-A71. Population per county as a function of the proportion of zeros in weekly incidence for observed and predicted time series. The intersection of the fitted function with the y-axis indicated the estimate of the critical community size for EV-A71. N is the number of counties with at least one epidemic fadeout.

**Figure 3:**
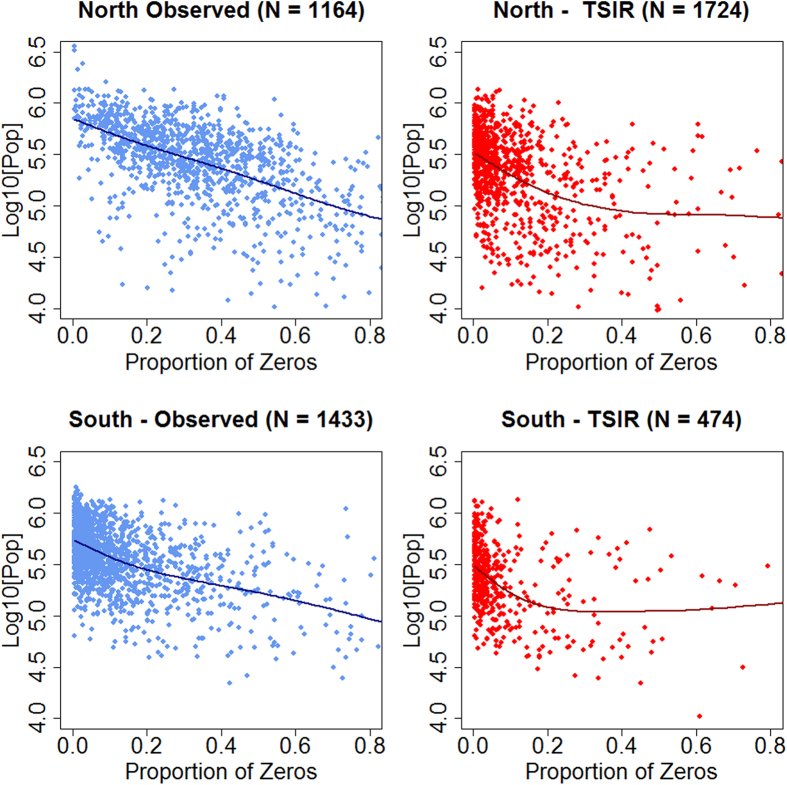
Critical community size of CV-A16. Population per county as a function of the proportion of zeros in weekly incidence for observed and predicted time series. The intersection the fitted function with the y-axis indicated the estimate of the critical community size for CV-A16. N is the number of counties with at least one epidemic fadeout.

Additionally, a conditional threshold of one weekly migrant was imposed during the minimization procedure in order to avoid biased values (overestimates) for 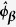 as a compensation mechanism for premature stochastic extinction in small counties. Using this condition for the minimization procedure did not substantially affect the within-county dynamics (Appendix V), as the number of infected migrants remained on average one order of magnitude lower than the observed incidence in the counties with the lowest HFMD presence (25% of total counties). When running simulations to estimate the CCS and test vaccination strategies this condition was relaxed in order to enable the extinction-recolonization dynamics. Finally, the simulated incidence obtained through this two-step procedure was used to calculate the CCS following the same methodology as described for the observed incidence time series (Fig. 2 for EV-A71 and Fig. 3 for CV-A16). Finally, the differences (or lack thereof) CCS obtained for each region, and incidence time series (observed *vs* predicted) was assessed using a three step procedure: fist we used the jackknife residuals of the fitted smooth spline to calculate the 95% CI around the intersection of the spline with the population axis. Second, based on this interval, we constructed normal distributions of the probable CCS value with a number of samples equal to the number counties used to estimate to CCS in each region. Third we used a two-sided t-tests on these distributions to assess the difference in CCS between the two regions. Two-sided t-tests weighted by the number of counties used for the estimation (Figs 2 and 3).

### Vaccination Strategies

In each county, simulations of vaccination were run using the scaling factors 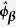, and 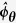, identified from the optimization procedure, as well as the initial number of infectious and susceptible individuals on the first week of the 39^th^ year of simulation. Epidemics were run for 20 additional years before vaccination started.

**Figure 4:**
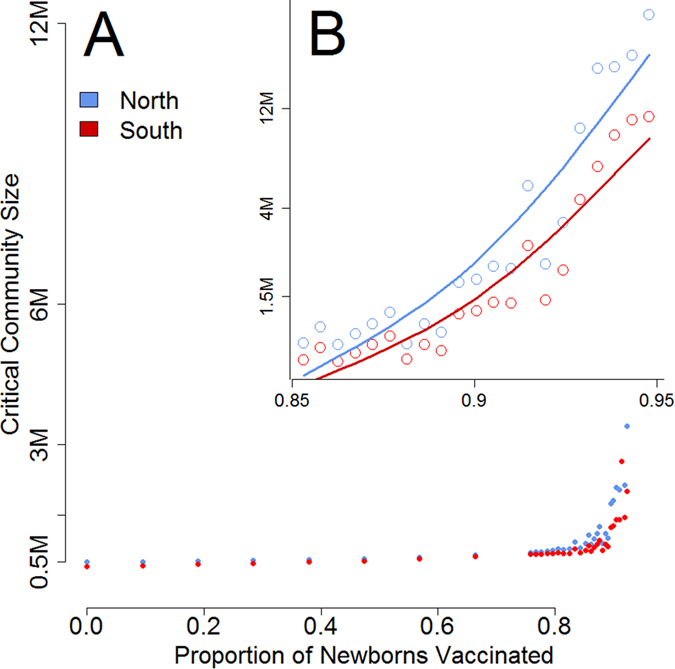
Routine vaccination. Effect of routine vaccination at birth on the critical community size of HFMD in the Northern (blue) and Southern (red) regions of China.

First, we studied the impact of routine vaccination of newborns with increasing coverage rate *p* on the CCS of HFMD in each epidemic region of China (Fig. 4).

Second, we considered the eventual targeting of a limited number of vaccines to newborns in ‘priority areas’, thus implementing spatially heterogeneous vaccination strategies. The outcome of each strategy is measured by the decrease in the cumulated number of cases 5 years and 20 years following vaccination. In this instance, for each week, a number of doses equal to half the number of births per week in 2013, (n = 101,596 individuals) was distributed across the country according to five spatial strategies: A) Vaccination targeted in counties with the lowest population (n = 1,601 locations), B) vaccination in counties with the highest population (n = 678 locations), C) vaccination targeted in the counties which population is the closest to the regional CCS (n = 1,155 locations), D) vaccination in randomly selected counties (n = 1,217 locations), and E) vaccination in counties with the highest average incidence (n = 1,669).

Third, we considered a case in which newborns across the entire country are vaccinated routinely with a coverage rate of 85%, in every county (strategy F). This value was chosen to reflect the typical coverage for vaccines included in China’s National Immunization Program. We compared this strategy with the five spatially explicit vaccination strategies: the total number of doses used for strategy F was targeted to specific counties according to a spatial strategy, A, B, C, D, or E. In each county selected for vaccination, 85% of newborn were vaccinated and, in addition, an equal number of doses was administered through a single pulse vaccination of susceptible individuals on the 6^th^ week of the year (which corresponds to the minimum of the yearly incidence). Thus for strategies A, B, C, D and E, 42.5% of newborn in the whole country received routine vaccination. But vaccination at birth only occurs in selected counties (with coverage 85%) and is complemented by pulse vaccination. In the counties selected for vaccination, Eq. 2 can be extended for each year as follows,


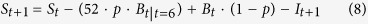


where the weekly number of birth *B*_*t*_ is shrunk by a factor (1-*p*) with *p* corresponding to the vaccination coverage at birth adjusted for vaccine efficacy (94.80%)^19^ and (*52· p·B*_*t|t*=6_) corresponds to the number of vaccine doses administered through pulse vaccination on the 6^th^ week of a given year, once a year. Finally, for routine vaccination of newborns, we also considered coverage rates reflecting differences in socio-economic status across provinces (strategy F*), see Appendix III). The provincial coverage rates were derived for three childhood vaccines (Haemophilus influenza type B vaccine, rotavirus vaccine and Varicella vaccine). These were obtained through a literature review and stratified across three socio-economic groups (high-, middle-, and low-income provinces). Each province was assigned a minimum, mean and maximum coverage rate based on the literature available in its corresponding socio-economic group. Because these vaccines and the current candidate vaccine against EV-A71 are not currently part of the National Immunization Program of China’s Ministry of Health, the uptake in each province may be influenced by local economic conditions^47^. All simulations of vaccination were replicated 250 times to compute confidence intervals through Monte Carlo simulations for the reduction of incidence after 5 and 20 years. The differences (or lack thereof) in the reduction of the number of infected individuals associated with each vaccination strategy was assed using an analysis of variance followed by multiple comparison test (Tukey).

## Results

### Model Fitting

For EV-A71, the median correlation coefficient and median R^2^ between observed and simulated incidence at the provincial level were respectively 0.88 and 0.78 (Table 1). For CV-A16, the median correlation coefficient and median R^2^ between observed and simulated incidence at the provincial level were respectively 0.90 and 0.81 (Table 2). A scaled TSIR model including the extinction-recolonization dynamics could be fitted in 2,897 counties. The incidence weighed correlation coefficient was 0.47, and the R^2^ was 0.27 for EV-A71 and 0.49 and 0.30 for CV-A16. The median reporting rates were very low, 4.20% for EV-A71 (Table 1) and 2.90% for CV-A16 (Table 2) respectively. The average transmission rates were consistent with the estimates of Takahashi *et al.*^38^ using data from NIDRIS.

### Critical Community Size

For EV-A71 (Fig. 2), in the northern region the CCS was 722,372 (±150,562 C.I. 95%) for the estimate obtained from the observed time series and 390,850 (±189,744 C.I. 95%) for the estimate obtained from the simulated time series. Whilst in the southern region, the estimates for the CCS were 525,104 (±279,791 C.I. 95%) for the estimates obtained from the observed time series and 336,979 (±225,866 C.I. 95%) for the estimate obtained from the simulated time series. For CA-61 (Fig. 3), in the northern region the CCS was 679,249 (±190,549 C.I. 95%) for the estimate obtained from the observed time series and 332,983 (±213,456 C.I. 95%) for the estimate obtained from the simulated time series. In the southern region, the estimates for the CCS were respectively 554,268 (±266,154 C.I. 95%) and 315,768 (±249,074 C.I. 95%) for the estimates obtained from the observed and simulated time series. We thus find comparable measures of CCS for CV-A16 and EV-A71. Significant differences in CCS were identified between regions (North versus South) when considering the observed incidence time series (p-value = <0.001 for both CV-A16 and EVA-71) but not when considering the simulated incidence for CV-A16 (p-value = 0.35). When considering the simulated incidence for EVA-71, a significant difference was identified between the northern and the southern region (p-value < 0.001) but its magnitude was limited compared with the difference in CCS obtained from comparing the observed incidence time series. Additional evidence for stronger persistence of EV-A71 in the southern region comes from the higher proportion of counties without a single epidemic fadeout in the southern region compared to the northern region. The southern region had an observed 21% and a predicted 62% of counties with no epidemic fadeout, while the northern region had less than 1% observed and 14% of predicted counties with no epidemic fadeout between 2010 and 2013.

### Vaccination Strategies

Simulations of routine vaccination of newborns across China suggest that the CCS would significantly increase at high coverage rates (Fig. 4A). At a vaccination rate of 85%–as expected if the vaccine is included in the NIP– the benefits of routine vaccination would be comparable in the northern and southern regions with increases in CCS of 130% (from 390,850 to 900,750) and 125% (from 336,979 to 758,332) respectively. Further increasing vaccination rates (>90%) to raise the CCS would marginally benefit the Northern region relative to the Southern region (Fig. 4B), this is that any increment of 1% in vaccination coverage yields to a higher increase in CCS in the northern region compared to the Southern region. However these estimates should be treated with caution; at high vaccinations rates (95–99%) there are only a few dozen counties experiencing discontinued epidemics that could be used in further simulations to estimate the CCS.

**Figure 5:**
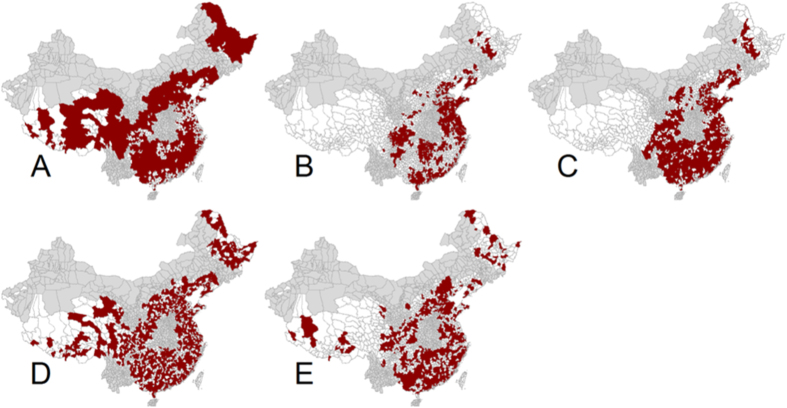
Spatial vaccination strategies. (**A**) Vaccination targeted in counties with the lowest population, (**B**) vaccination in the highly populated counties, (**C**) Vaccination in the counties where population is the close to the regional CCS, and (**D**) vaccination in randomly selected counties. (**E**) Vaccination in high prevalence counties. Red indicates vaccinated areas, white indicates unvaccinated areas, grey indicates areas excluded from the analysis. This figure was generated using the open source statistical software R (cran.r-project.org), version number 3.0.3, including packages maptools and foreign.

Five spatial vaccination strategies were simulated at the county level (Fig. 5, Tables 3 and 4). For Strategy A, large rural counties in the western region of China, Sichuan and Heilongjiang provinces as well as the peri-urban counties of the South East were selected for vaccination. All major agglomerations were excluded. For Strategy B areas subject to vaccination are located in major urban center such as the south of Guangdong province, the municipalities of Shanghai and Beijing, as well as the surroundings of Chongqing, Chengdu and Hunan provinces. For strategy C most peri-urban areas in the country were selected for vaccination, but all the very rural or highly populated counties located on the South-East coast were excluded. For strategy E most urban centers were selected for vaccination with additional counties in the northern and western region.

In a context where the number of doses available is equal to half the number of yearly births (Table 3), the strategy consisting in targeting vaccination in areas of highest prevalence (E) or the largest population (C) showed the largest reduction in the cumulative number of infectious individuals over a period of 5-years (E = 48.8%, C = 46.7%) and 20-years (E = 53.8%,C = 49.5%). Compared with strategies A, B and D, strategy (E) was 37.8% (A), 32.2% (B) and 35.1% (D) more efficient for reducing incidence five years after vaccination, and (A) 26.8% , (B) 23.1%, and (D) 24.2% 20-years after vaccination (Appendix VI).

In a context where the number of doses available is sufficient to achieve 85% coverage of newborns, this vaccination strategy (F) outperforms all the spatially explicit strategies based on a combination of newborn vaccination and pulse vaccination using the same number of doses across the country (A–E). The routine vaccination of newborns at coverage rates reflecting the level of coverage achievable under the national immunization program could result in a reduction in the number of infectious individuals of 71.7% after 5 years and 77.8% after 20 years (Table 4). The relative reduction in the number of infected individuals after 5 years for routine vaccination was 92% to 47% more important compared with all the spatially targeted strategies combining routine birth vaccination with pulse vaccination. Finally, the routine vaccination of newborns with coverage rates reflecting vaccine uptake for other similar childhood diseases that are not currently included in the NIP (strategy F*) showed a low reduction in the number of infectious individuals (40% after five years) compared to the reduction that could be achieved under the NIP (>85% coverage).

## Discussion

### Critical Community Size

In this study we used incidence time series and mathematical models to estimate the CCS of HFMD in China, and investigate the effect of spatially heterogeneous vaccination strategies against EV-A71. To our knowledge this study is the first to attempt to quantify the CCS of HFMD in China. Our results suggest a comparable CCS for CV-A16 and EV-A71 but significant differences between regions (North versus South). The magnitude of these regional differences, and in particular the smaller critical community sizes identified in the southern region are consistent with the longer seasonal persistence reported by Xing *et al.*^12^ as well as and the bi-annual transmission dynamics identified by Takahashi *et al.*^38^. Longer transmission seasons in the South potentially allows the disease to persist in smaller communities by increasing chances of re-introduction, and this may be a critical feature to consider when implementing nation-wide vaccination campaigns. In this context, we identified that routine vaccination of newborns could substantially increase the population threshold for persistence of HFMD. The benefits of routine vaccination would be more pronounced in the northern region at moderate vaccination rates. Overall, we found considerable evidence for local persistence of both serotypes of HFMD, in particular in the southern region.

For each strain, EV-A71 and CV-A16, we found important discrepancies between CCS estimates obtained through simulation versus reported incidence, with ratios ranging from 2.03:1 to 1.56:1 depending on the serotypes and region considered. The smaller regional differences for the CCS obtained from the simulated incidence may further be attributed to this procedure because by indistinctively filling interruptions in times series the process may generate similar epidemic patterns from small outbreaks. CCS estimates obtained from the reported cases might at first be considered more reliable, as they are directly derived from only epidemiological evidence. However, we show that the reporting rates of HFMD are likely very low in agreement with previous evidence for underreporting^38^. The proportion of zeros in the incidence time series may thus be largely overestimated and consequently lead to overestimates of CCS^28^. Whilst our approach for correcting underreported time series partly attempts to address this issue (Appendix II), the adjusted incidence should nonetheless be treated with caution. As a consequence of the very low reporting rates, prolonged interruptions in reported incidence may correspond to either an actual interruption in transmission or no cases being reported. In the absence of detailed epidemiological studies on treatment-seeking behavior as well as a better quantification of potentially time varying reporting rates these two situations cannot be distinguished. In this context CCS estimates obtained from simulated incidence provide a methodological alternative to the correction of observed incidence with very low and therefore uncertain reporting rates. Additionally, the smaller regional differences for the CCS obtained from the simulated incidence may further be attributed the underreporting procedure and the migration terms as implemented in the TSIR model, because by indistinctively filling interruptions in times series the process may generate similar epidemic patterns for different areas characterized by regular small outbreaks. Whilst the accuracy of the estimates obtained through simulation is contingent on the parameterization of the immigration process as well as the mathematical structure of our epidemic model, we found that our estimates of CCS are not dissimilar from other (and more extensively studied) self-immunizing childhood illnesses such as measles , rubella and pertussis^29^.

### Spatial Vaccination Strategies

We simulated seven vaccination strategies, including five strategies to target a limited number of doses to specific geographic areas characterized by either high, low, medium and randomly selected population sizes or high prevalence of HFMD. The underlying and competing hypothesis as to why each vaccination strategy implemented could yield to the best results in term of reducing the number of HFMD cases goes as follows. On one hand, targeting vaccination primarily in highly populated (or highly infected) counties could decrease the intensity of the recolonization mechanism even if the level of vaccination is insufficient to locally eradicate the disease. This is possible because highly populated (or highly infected) counties contribute an important share to the provincial incidence which directly affects the intensity of the recolonization process as formulated in our model. This strategy could be seen as ‘turning off the tap’ of infections from large areas that might subsequently spillover into the smaller areas. On the other hand, the alternative hypothesis which consists of targeting vaccination primarily in many small populations is related to the idea that if the small populations are brought under a critical threshold, the probability of stochastic extinction at the scale of small populations increases substantially, and thus epidemics will progressively fade out as the result of susceptible depletion. An analogy for this strategy could be seen as ‘putting out a large fire with a blanket’, working from the edge to limit the oxygen uptake and progressing towards the center of the fire. In this study, we observe that the ‘turning off the tap’ strategies—targeting vaccination in high prevalence areas or large populations—outperforms the other strategies implemented. Vaccination targeted in populations close to the CCS does not have a strong advantage over vaccination in small areas^43^. Thus if vaccination campaigns against HFMD should be implemented in the foreseeable future, and if the number of doses is limited, our results suggest these resources would be optimally deployed in highly populated urban areas and in high prevalence areas which is potentially also easier to implement from a logistical standpoint. If the number of doses is sufficient to cover all new births, our results suggest that routine vaccination at birth would be the most effective allocation of vaccines provided that the vaccine is included in the NIP, and reaches high coverage level (>85%). All spatially heterogeneous vaccination strategies based on a combination of pulse vaccination and birth vaccination were largely outperformed by routine vaccination with an equal number of doses by a large margin.

### Limitations

As with any model-based study, our analysis is subject to assumptions and limitations. The TSIR framework used in this study does not presently account for age structure, cross-protection between HFMD serotypes or waning of vaccine-induced immunity (Hsiao *et al.*, in prep^45^,). Our estimates for the reduction in the number of cases after 5 and 20 years may thus represent a conservative overestimate of the time necessary to reach the low levels of incidence presented in this study. Additionally the models used here were parameterized using the last four years of the time series of HFMD incidence. This choice was motivated by the unreliable reporting of HFMD in 2008–2009 that resulted in poor statistical fits at the province level if reports prior to 2010 were included. Using a 4-year time series here is necessarily short compared to other studies using the TSIR framework, and this may thus affect the robustness of our simulations. Additionally, in order to fit epidemics parameters in each county we used a scaling methodology that involves a baseline immigration of one migrant per week. Whilst this has limited effect on the overall dynamics of HFMD within each spatial unit (Supplementary Fig. S4) it may have marginally affected our final estimates of CCS. Finally, as surveillance has improved over the last decade, more recent incidence reports may be associated with higher reporting rates^48^, although yearly variation is partially captured through the use of a time-varying function such as the smooth spline used in the study.

### Conclusion and next steps

Over the last couple of decades seasonal HFMD epidemics occurred regularly in East and South East Asian countries. Over the course of this period, urban migrations and urban population in these regions have increased in an unprecedented way (World Bank 2015). Symptomatic of this trend is the fact that 4 of the world’s 10 largest cities in 2015^49^ were located in China. In this study, we highlighted the impact of such densely populated areas on disease dynamics: first these densely populated areas allow a disease such as HFMD to persist over large geographic areas despite its relatively high CCS, and second these areas play a key role in sustaining the immigration-recolonization dynamics in less densely populated peri-urban areas. Further work on the timing, seasonality and synchrony of epidemics along an urbanization gradient could help better understand the spatio-temporal dynamics of HFMD as well as other emerging childhood diseases in Asia. In this context, data on urbanization and within-China population movements could help to shed light on the mechanistic process underlying the apparent dynamics of numerous childhood diseases.

## Additional Information

**How to cite this article**: Van Boeckel, T. P. *et al.* Hand, Foot, and Mouth Disease in China: Critical Community Size and Spatial Vaccination Strategies. *Sci. Rep.* **6**, 25248; doi: 10.1038/srep25248 (2016).

## Supplementary Material

Supplementary Information

## Figures and Tables

**Table 1:** EV71.

Province	Corr.	R^2^	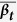	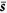	Rep. Rate
Beijing	0.792	0.628	42.74	0.035	0.0995
Tianjin	0.925	0.856	6.41	0.232	0.0897
Hebei	0.954	0.91	37.87	0.043	0.0391
Shanxi	0.969	0.939	35.58	0.044	0.0338
Inner Mongolia	0.926	0.857	2.09	0.738	0.03
Liaoning	0.875	0.766	35.25	0.043	0.0607
Jilin	0.932	0.868	5.71	0.262	0.0432
Heilongjiang	0.946	0.894	15.11	0.101	0.0186
Shanghai	0.864	0.746	22.8	0.067	0.0914
Jiangsu	0.907	0.823	30.12	0.054	0.0633
Zhejiang	0.881	0.776	43.16	0.037	0.0973
Anhui	0.868	0.754	36.99	0.044	0.0559
Fujian	0.887	0.786	52.61	0.03	0.0743
Jiangxi	0.816	0.666	20.16	0.08	0.0282
Shandong	0.975	0.95	5.31	0.308	0.0418
Henan	0.937	0.878	8.29	0.2	0.0296
Hubei	0.935	0.875	12.23	0.132	0.0544
Hunan	0.847	0.718	26.14	0.063	0.0657
Guangdong	0.863	0.746	19.65	0.084	0.1144
Guangxi	0.853	0.728	21.28	0.076	0.125
Hainan	0.723	0.523	11.32	0.131	0.1594
Chongqing	0.856	0.732	104.05	0.015	0.0355
Sichuan	0.799	0.639	96.06	0.017	0.0269
Guizhou	0.834	0.695	28.37	0.056	0.0405
Yunnan	0.886	0.785	47.14	0.034	0.0401
Tibet	0.617	0.38	30.61	0.049	0.0118
Shaanxi	0.932	0.869	55.65	0.028	0.0641
Gansu	0.97	0.941	20.41	0.076	0.0153
Qinghai	0.617	0.38	8.26	0.187	0.0111
Ningxia	0.972	0.945	1.47	1	0.0373
Xinjiang	0.965	0.932	4.7	0.331	0.0092

Provincial TSIR, fitting correlation coefficient, coefficient of determination, mean transmission rate and mean proportion of susceptible.

**Table 2:** CA16 Provincial.

Province	Corr.	R^2^	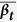	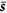	Rep.Rate
Beijing	0.907	0.823	50.08	0.03	0.0658
Tianjin	0.799	0.639	29.17	0.051	0.06176
Hebei	0.959	0.92	28.63	0.057	0.02432
Shanxi	0.925	0.856	35.63	0.044	0.02349
Inner Mongolia	0.937	0.878	25.28	0.061	0.02536
Liaoning	0.959	0.92	33.73	0.045	0.03746
Jilin	0.937	0.878	31.11	0.048	0.02974
Heilongjiang	0.516	0.266	66.61	0.023	0.01076
Shanghai	0.85	0.722	37.21	0.041	0.07835
Jiangsu	0.928	0.862	30.68	0.053	0.03842
Zhejiang	0.878	0.771	24.2	0.066	0.05809
Anhui	0.86	0.74	23.62	0.069	0.03612
Fujian	0.892	0.796	21.36	0.074	0.04757
Jiangxi	0.827	0.684	17.02	0.095	0.01899
Shandong	0.977	0.954	11.93	0.137	0.0273
Henan	0.934	0.872	36.02	0.046	0.01834
Hubei	0.917	0.84	29.91	0.054	0.03221
Hunan	0.872	0.76	29.91	0.055	0.04104
Guangdong	0.842	0.708	56.93	0.029	0.07558
Guangxi	0.857	0.734	40.41	0.04	0.08797
Hainan	0.712	0.507	21.83	0.068	0.08981
Chongqing	0.87	0.757	16.91	0.093	0.02302
Sichuan	0.812	0.66	29.24	0.056	0.01494
Guizhou	0.805	0.648	26.06	0.061	0.02541
Yunnan	0.88	0.775	23.24	0.069	0.02726
Tibet	0.458	0.21	11.15	0.143	0.00595
Shaanxi	0.963	0.927	41.23	0.038	0.03663
Gansu	0.949	0.9	34.35	0.045	0.00997
Qinghai	0.845	0.715	5.22	0.296	0.03849
Ningxia	0.946	0.894	10.32	0.143	0.02454
Xinjiang	0.935	0.873	28.09	0.056	0.00502

TSIR fitting correlation coefficient, coefficient of determination, mean transmission rate and mean proportion of susceptible.

**Table 3:** Mean reduction in the cumulated number of infectious individuals following weekly vaccination of 50% of newborns according to different spatial vaccination strategies.

Spatial Strategy	Red 5-Years (%) ± 95%	Red 20-Years (%) ± 95%
Random (A)	35.4 [33.1;37.7]	42.4 [41.9;42.8]
Close-CCS (B)	36.9 [34.2;39.6]	43.7 [43.3;44.2]
Large(C)	46.7 [44.2;49.2]	49.5 [49.0;50.1]
Small(D)	36.1 [33.7;38.5]	43.3 [42.8;43.7]
High Prevalence (E)	48.8 [46.7;50.9]	53.8 [53.4;54.2]

Coverage rate = 85% and vaccine efficacy = 94.8% in each vaccinated county.

**Table 4:** Reduction in the cumulated number of infectious individuals following vaccination of 85% newborns at birth (strategy F) or combination of routine vaccination (42.5%) and pulse vaccination (42.5%) according to different spatial vaccination strategies (A, B, C, D, E).

Strategy	Red 5-Years (%) ± 95%	Red 20-Years (%) ± 95%
Random (A)	46.1 [44.0;48.2]	48.1 [47.7;48.5]
Close-CCS (B)	38.2 [35.7;40.7]	43.3 [42.8;43.8]
Large(C)	47.7 [45.1;50.2]	49.2 [45.5;52.9]
Small(D)	37.3 [35.2;39.5]	42.4 [42.0;42.9]
High Prevalence (E)	48.6 [46.3;50.8]	52.4 [52.0;52.9]
Routine 85% (F)	71.7 [70.0;73.4]	77.8 [77.3;78.2]
Routine non-NIP (F*)	39.4 [23.3;56.6]	40.7 [25.2;60.0]

Coverage rate = 85% and vaccine efficacy = 94.8% in each vaccinated county.

^*^The confidence interval for simulations of vaccination outside of *China’s National Immunization Program* (non-NIP) are based on the range of estimates found from the existing literature (Supplementary Information, Table SIV 1).
