# Supplementary material for: Hand, Foot, and Mouth Disease in China: Critical Community Size and Spatial Vaccination Strategies
Source: Sci Rep. 2016 Apr 29;6:25248. doi: 10.1038/srep25248 (PMC4850478; doi:10.1038/srep25248)
Supplement: Supplementary Information [file srep25248-s1.pdf]

Supplementary Information

Hand, foot, and mouth Disease in China: Critical Community Size and Spatial Vaccination Strategies

Authors: Thomas P. Van Boeckel[1,3], Saki Takahashi[1], Qiaohong Liao[2], Weijia Xing[2], Shengjie Lai[2,6], Victor Hsiao[1], Fengfeng Liu[2], Yaming Zheng[2], Zhaorui Chang[2], Chen Yuan[2], C. Jessica E. Metcalf[1,5], Hongjie Yu[2*], and Bryan T. Grenfell[1,4*].

Affiliations:

1. Department of Ecology and Evolutionary Biology, Princeton University, Princeton, NJ, USA

2. Division of Infectious Diseases, Key Laboratory of Surveillance and Early–warning on Infectious Disease, Chinese Center for Disease Control and Prevention, Beijing, China

3. Institute of Integrative Biology, ETH Zurich, Zurich, Switzerland

4. Fogarty International Center, National Institutes of Health, Bethesda, MD, USA

5. Woodrow Wilson School of Public and International Affairs, Princeton University, Princeton, New Jersey, USA

6. Department of Geography and Environment, University of Southampton, Southampton, UK

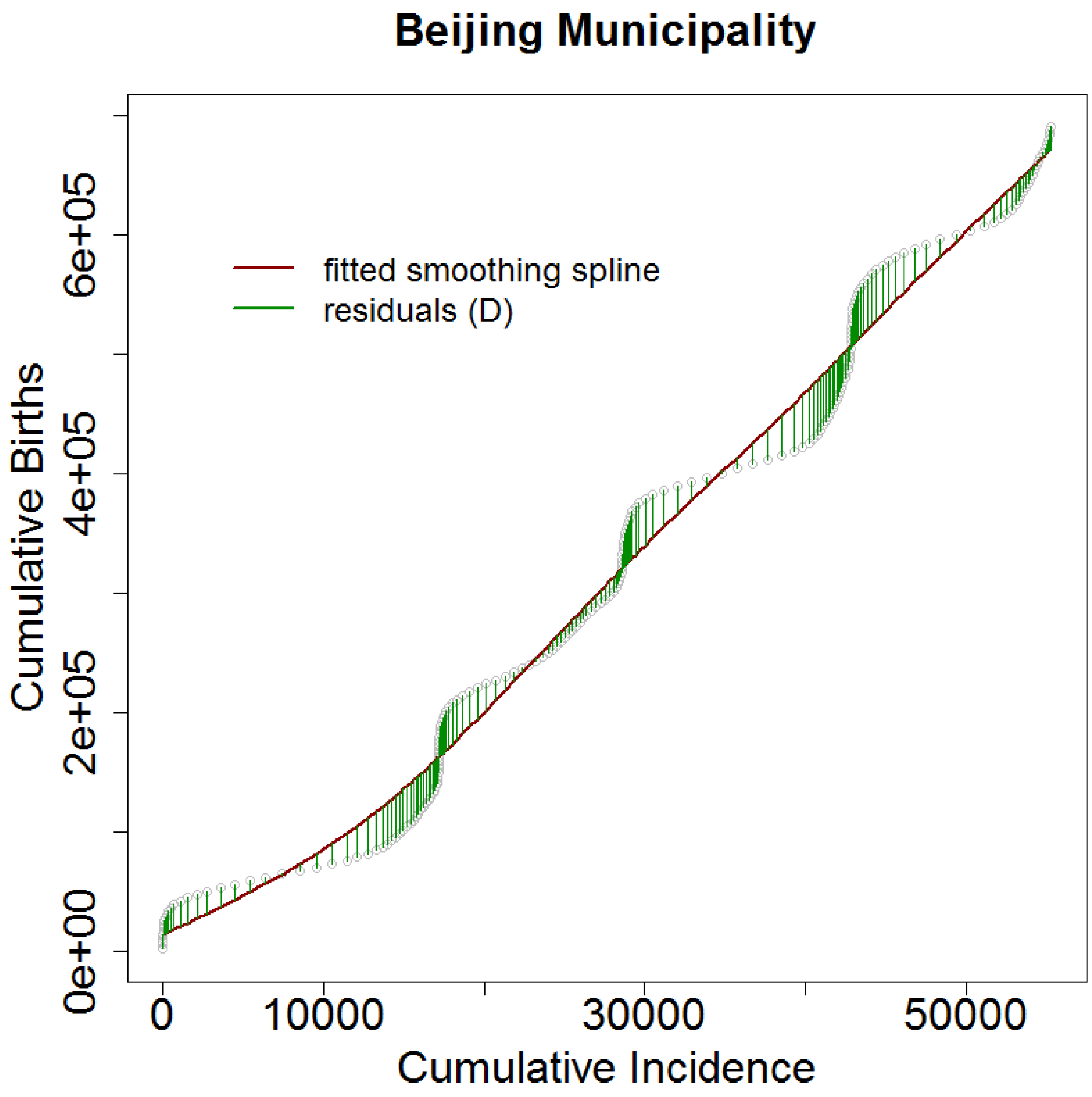

Supplementary Figure S1*. The weekly reporting rates were estimated in each province as the inverse of the slope of the locally varying regression (red, smoothing spline, df = 2.5) of the cumulative number of births over the cumulative incidence. The residuals of the relationship were used to modulate the amplitude of the variation around the mean number of susceptible to reconstruct the time series of susceptible in each province.*

Appendix II. Correcting for underreporting

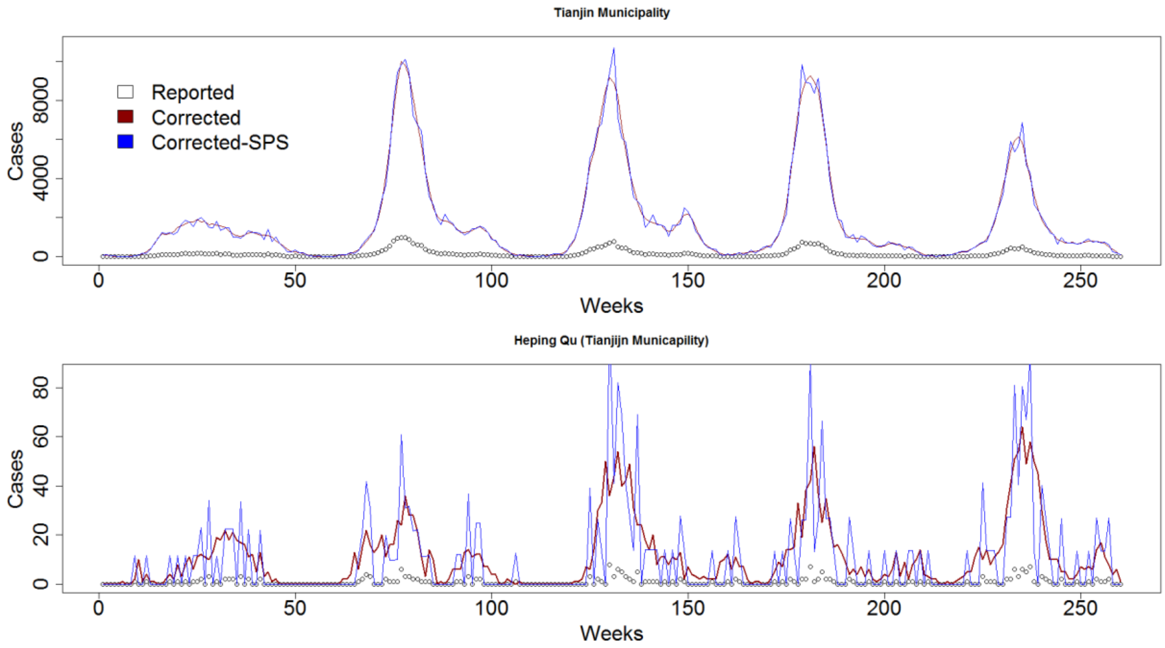

Supplementary Figure S2*. Correction of HFMD incidence time series for under reporting*

After evaluating the underreporting rates in each province we corrected the reported incidence time series in each county using a function described below and referred to on the figure as Corrected-SPS for *Smoothing and Poisson Sampling*. The function takes as arguments (TSs) the incidence time series and its associated reporting rates (RR).

```
function(TSs,RR){
  mySSTSM = smooth.spline(1:length(TSs), TSs)$y
  mySSTSMSum = mySSTSM * RR
  mySSTSMSum[mySSTSMSum<0] = 0
  PoisConstruct = rpois(length(mySSTSM),mySSTSMSum)
return(PoisConstruct)
}
```

This type of correction has two advantages over a mere multiplication by the inverse of the reporting rate when correcting for small populations. First, this corrective function better captures the tails of each epidemic wave, which potentially reduces the proportion of null weeks in the times series in the context of evaluating the critical community size. Second, this reduces the overall stochasticity of the signal which facilitates any posterior model fitting procedure such as the scaling of transmission rates.

Appendix III. Provincial vaccine coverage rates for EV-A71 derived from economic status

In order to generate plausible estimates and confidence intervals for the future coverage rates of the EV-A71 vaccine, we used estimates obtained from the literature for other vaccines that are not currently included in the national immunization program (referred to as 'type 2 vaccines' in China). In each province, we used an average of the coverage rates associated with the following vaccines as proxies: Haemophilus influenza type B vaccine (Hib), rotavirus vaccine (ROV) and Varicella vaccine (VAR).

Because these vaccines are not currently part of the National Immunization Program, uptake is closely associated with the economic development level in each province. Provinces were categorized in three groups of economic development according to their GDP per capita with respectively category 1 > 60000 CNY; category 2, 60,000-40,000 CNY; and category 3, < 40,000.

To obtain provincial coverage rates for those diseases, we searched for published studies on China using the keywords "coverage rate" in Pubmed, CNKI and the Wanfang database. A total of 21 studies could be found, and the following information was extracted: year of study, age of study, population size, study site, and coverage rate (minimum, mean and maximum).

Coverage rates for PCV-7 were not included in this study because the vaccine was not supplied in China in 2013-14. Coverage rates for InfV were not included because the target population for this vaccine is primarily adults and elderly people. Coverage rates for other vaccines that did not involve children were excluded from the data gathering process. Provincial coverage rates for the listed diseases (Hib, ROV, VAR) were obtained for the following provinces: Chongqing, Guangdong, Zhejiang, Henan, Jiangsu, Beijing as well as for the national average. When a coverage rate could not be obtained for a specific province, this province was assigned the average, minimum and maximum coverage rate of its corresponding economic development group. Coverage rates for two national level studies were assigned associated with the middle-income category (category 2), because coverage rates were not available for different categories of income.

Supplementary Table SIII 1. Provincial vaccine coverage rate for Hib, Var and ORV in China

Category	Province	GDP per capita in 2014 (CNY)	Haemophilus influenzae type b (Hib)		Varicella (Var)		Rotavirus (ORV)	
			high	low	high	low	high	low
1	Tianjin	105202	91.6%	36.2%	90.3%	19.4%	89.9%	27.5%
	Beijing	99995	91.6%	36.2%	90.3%	19.4%	89.9%	27.5%
	Shanghai	97343	91.6%	36.2%	90.3%	19.4%	89.9%	27.5%
	Jiangsu	81874	91.6%	36.2%	90.3%	19.4%	89.9%	27.5%
	Zhejiang	72967	91.6%	36.2%	90.3%	19.4%	89.9%	27.5%
	Neimenggu	71044	91.6%	36.2%	90.3%	19.4%	89.9%	27.5%
	Liaoning	65201	91.6%	36.2%	90.3%	19.4%	89.9%	27.5%
	Fujian	63472	91.6%	36.2%	90.3%	19.4%	89.9%	27.5%
	Guangdong	63452	91.6%	36.2%	90.3%	19.4%	89.9%	27.5%
	Shandong	60879	91.6%	36.2%	90.3%	19.4%	89.9%	27.5%
2	Jilin	50162	69.7%	43.0%	44.7%	18.0%	31.0%	17.0%
	3chongqing	47859	69.7%	43.0%	44.7%	18.0%	31.0%	17.0%
	Hubei	47124	69.7%	43.0%	44.7%	18.0%	31.0%	17.0%
	Shaanxi	46929	69.7%	43.0%	44.7%	18.0%	31.0%	17.0%
	Ningxia	41834	69.7%	43.0%	44.7%	18.0%	31.0%	17.0%
	Xinjiang	40607	69.7%	43.0%	44.7%	18.0%	31.0%	17.0%
	Hunan	40287	69.7%	43.0%	44.7%	18.0%	31.0%	17.0%
3	Hebei	39984	46.4%	39.2%	42.4%	23.3%	24.5%	9.6%
	Qinghai	39633	46.4%	39.2%	42.4%	23.3%	24.5%	9.6%
	Heilongjiang	39226	46.4%	39.2%	42.4%	23.3%	24.5%	9.6%
	Hainan	38924	46.4%	39.2%	42.4%	23.3%	24.5%	9.6%
	Henan	37073	46.4%	39.2%	42.4%	23.3%	24.5%	9.6%
	Sichuan	35128	46.4%	39.2%	42.4%	23.3%	24.5%	9.6%
	Shanxi	35064	46.4%	39.2%	42.4%	23.3%	24.5%	9.6%
	Jiangxi	34661	46.4%	39.2%	42.4%	23.3%	24.5%	9.6%
	Anhui	34427	46.4%	39.2%	42.4%	23.3%	24.5%	9.6%
	Guangxi	33090	46.4%	39.2%	42.4%	23.3%	24.5%	9.6%
	Xizang	29252	46.4%	39.2%	42.4%	23.3%	24.5%	9.6%
	Yunnan	27264	46.4%	39.2%	42.4%	23.3%	24.5%	9.6%
	Gansu	26427	46.4%	39.2%	42.4%	23.3%	24.5%	9.6%
	Guizhou	26393	46.4%	39.2%	42.4%	23.3%	24.5%	9.6%

Appendix IV. Sensitivity of the smoothing spline algorithm for determining the CCS

Supplementary Table IV 1

Variations in the estimates of the Critical Community Size of HFMD obtained from observed and simulated (TSIR) incidence according to the degree of freedom (df) used for the smoothing function (Figure 1 & 2).

df	Observed North	TSIR North	Observed South	TSIR South
1.5	695,976	336,091	473,868	288,674
2.5	707,758	358,481	486,160	304,689
3.5	727,488	388,039	509,206	330,289
4.5	762,186	398,968	525,458	342,749
5.5	816,342	405,497	535,146	346,020
6.5	875,596	412,474	541,798	346,074
CoV (sd/mean)	9.1%	7.7%	5.3%	7.4%

Appendix V. Conditional migration for transmission at the county level.

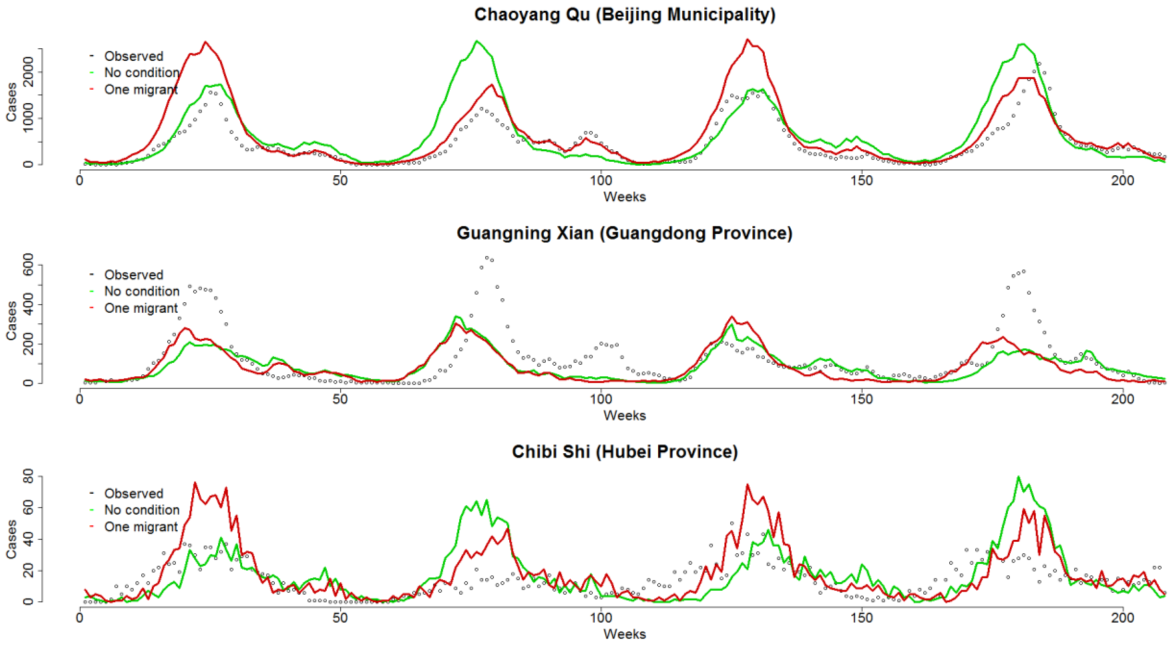

Supplementary Figure V1. *Comparison of simulation of the TSIR model at the county level with (red) and without (green) a constant immigration of at least one infected individual per week in three Chinese Provinces. Releasing the assumption on the constant influx of at least one migrant does not affect significantly the overall dynamics of HFMD even in counties with low population. For the 25% counties with the lowest incidence the mean incidence was one order of magnitude larger than the threshold of one migrant per week that was imposed for scaling the transmission parameters calculated at the provincial level by Takahashi et al.*

Appendix VI. Vaccination Strategies – Confidence Intervals

Reduction after 5 years for the cumulated number of infectious individuals following weekly vaccination of 50% of newborns according to different spatial vaccination strategies (coverage rate = 85% and vaccine efficacy = 94.8% in each vaccinated county)

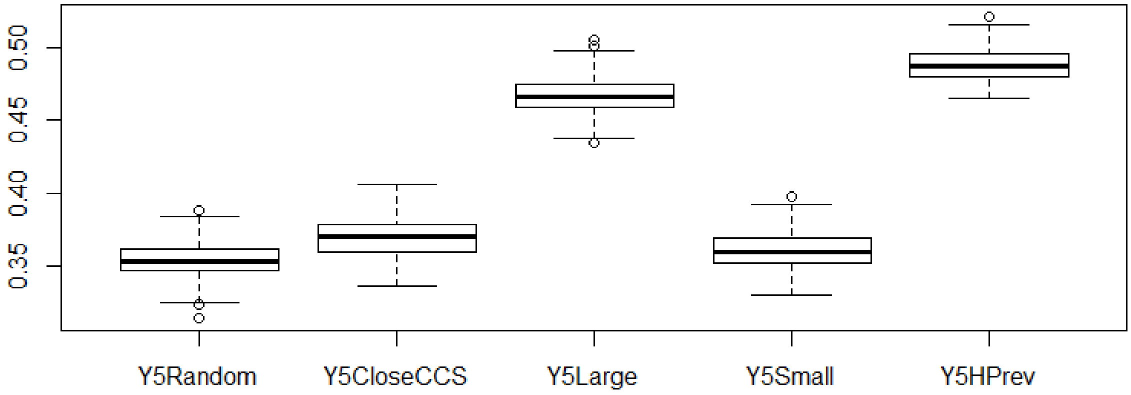


```
    Tukey multiple comparisons of means
    95% family-wise confidence level

Fit: aov(formula = reduction ~ factor(strategy), data = RRedsDF5Y)

$`factor(strategy)`
                            diff          lwr          upr p adj
Y5HPrev-Y5CloseCCS  -0.118915074 -0.121919608 -0.115910539     0
Y5Large-Y5CloseCCS  -0.097622102 -0.100626637 -0.094617567     0
Y5Random-Y5CloseCCS  0.015667141  0.012662607  0.018671676     0
Y5Small-Y5CloseCCS   0.008497638  0.005493104  0.011502173     0
Y5Large-Y5HPrev      0.021292972  0.018288437  0.024297506     0
Y5Random-Y5HPrev     0.134582215  0.131577680  0.137586750     0
Y5Small-Y5HPrev      0.127412712  0.124408177  0.130417247     0
Y5Random-Y5Large     0.113289244  0.110284709  0.116293778     0
Y5Small-Y5Large      0.106119740  0.103115206  0.109124275     0
Y5Small-Y5Random    -0.007169503 -0.010174038 -0.004164968     0
```

Reduction after 20 years for the cumulated number of infectious individuals following weekly vaccination of 50% of newborns according to different spatial vaccination strategies (coverage rate = 85% and vaccine efficacy = 94.8% in each vaccinated county)

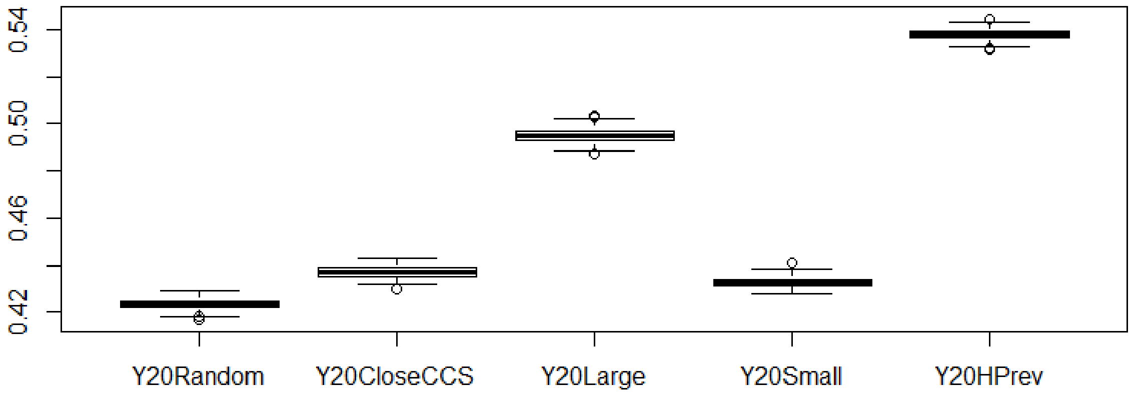


```
  Tukey multiple comparisons of means
    95% family-wise confidence level

Fit: aov(formula = reduction ~ factor(strategy), data = RRedsDF20Y)

$`factor(strategy)`
                              diff          lwr         upr p adj
Y20HPrev-Y20CloseCCS  -0.100779752 -0.101363727 -0.10019578     0
Y20Large-Y20CloseCCS  -0.058030848 -0.058614824 -0.05744687     0
Y20Random-Y20CloseCCS  0.013694241  0.013110265  0.01427822     0
Y20Small-Y20CloseCCS   0.004504055  0.003920080  0.00508803     0
Y20Large-Y20HPrev      0.042748904  0.042164928  0.04333288     0
Y20Random-Y20HPrev     0.114473993  0.113890017  0.11505797     0
Y20Small-Y20HPrev      0.105283807  0.104699832  0.10586778     0
Y20Random-Y20Large     0.071725089  0.071141114  0.07230906     0
Y20Small-Y20Large      0.062534903  0.061950928  0.06311888     0
Y20Small-Y20Random    -0.009190185 -0.009774161 -0.00860621     0
```

Reduction after 5 years in the cumulated number of infectious individuals following vaccination of 85% newborns at birth (strategy F) or combination of routine vaccination (42.5%) and pulse vaccination (42.5%) according to different spatial vaccination strategies (A, B, C, D, E). Coverage rate = 85% and vaccine efficacy = 94.8% in each vaccinated county.

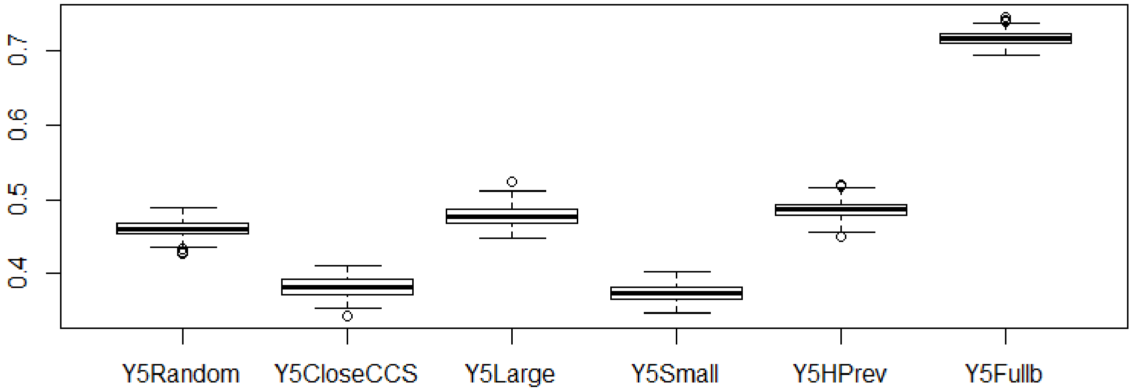


```
  Tukey multiple comparisons of means
    95% family-wise confidence level

Fit: aov(formula = reduction ~ factor(strategy), data = RRedsDF5Y)

$`factor(strategy)`
                           diff          lwr         upr p adj
Y5Fullb-Y5CloseCCS  -0.334919945 -0.337836772 -0.33200312     0
Y5HPrev-Y5CloseCCS  -0.103633830 -0.106550656 -0.10071700     0
Y5Large-Y5CloseCCS  -0.094713878 -0.097630705 -0.09179705     0
Y5Random-Y5CloseCCS -0.078964822 -0.081881648 -0.07604799     0
Y5Small-Y5CloseCCS   0.008649936  0.005733109  0.01156676     0
Y5HPrev-Y5Fullb      0.231286116  0.228369289  0.23420294     0
Y5Large-Y5Fullb      0.240206067  0.237289241  0.24312289     0
Y5Random-Y5Fullb     0.255955124  0.253038297  0.25887195     0
Y5Small-Y5Fullb      0.343569881  0.340653054  0.34648671     0
Y5Large-Y5HPrev      0.008919952  0.006003125  0.01183678     0
Y5Random-Y5HPrev     0.024669008  0.021752181  0.02758583     0
Y5Small-Y5HPrev      0.112283766  0.109366939  0.11520059     0
Y5Random-Y5Large     0.015749056  0.012832230  0.01866588     0
Y5Small-Y5Large      0.103363814  0.100446987  0.10628064     0
Y5Small-Y5Random     0.087614758  0.084697931  0.09053158     0
```

Reduction after 20 years in the cumulated number of infectious individuals following vaccination of 85% newborns at birth (strategy F) or combination of routine vaccination (42.5%) and pulse vaccination (42.5%) according to different spatial vaccination strategies (A, B, C, D, E). Coverage rate = 85% and vaccine efficacy = 94.8% in each vaccinated county.

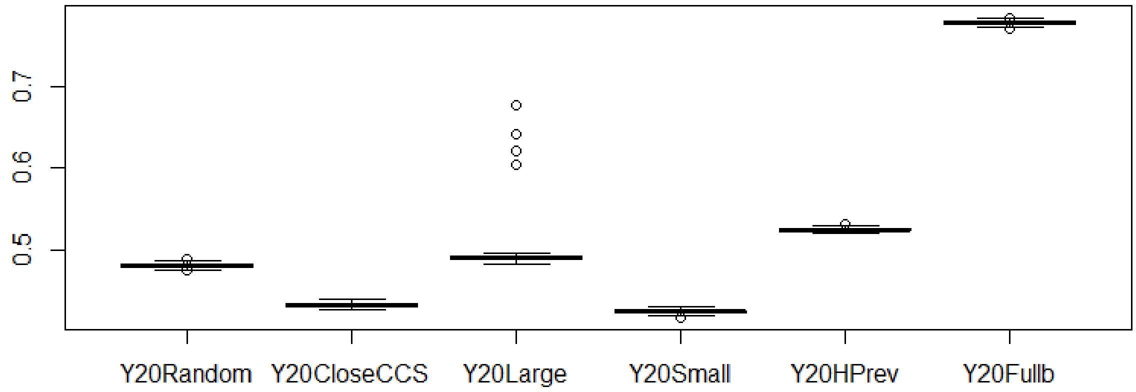


```
  Tukey multiple comparisons of means
    95% family-wise confidence level

Fit: aov(formula = reduction ~ factor(strategy), data = RRedsDF20Y)

$`factor(strategy)`
                              diff          lwr         upr p adj
Y20Fullb-Y20CloseCCS  -0.344735415 -0.346774313 -0.34269652     0
Y20HPrev-Y20CloseCCS  -0.091648619 -0.093687516 -0.08960972     0
Y20Large-Y20CloseCCS  -0.058998233 -0.061037130 -0.05695934     0
Y20Random-Y20CloseCCS -0.048305859 -0.050344757 -0.04626696     0
Y20Small-Y20CloseCCS   0.008685931  0.006647034  0.01072483     0
Y20HPrev-Y20Fullb      0.253086796  0.251047899  0.25512569     0
Y20Large-Y20Fullb      0.285737183  0.283698285  0.28777608     0
Y20Random-Y20Fullb     0.296429556  0.294390659  0.29846845     0
Y20Small-Y20Fullb      0.353421347  0.351382449  0.35546024     0
Y20Large-Y20HPrev      0.032650386  0.030611489  0.03468928     0
Y20Random-Y20HPrev     0.043342760  0.041303862  0.04538166     0
Y20Small-Y20HPrev      0.100334550  0.098295653  0.10237345     0
Y20Random-Y20Large     0.010692373  0.008653476  0.01273127     0
Y20Small-Y20Large      0.067684164  0.065645267  0.06972306     0
Y20Small-Y20Random     0.056991791  0.054952893  0.05903069     0
```
